# Associations of cerebral perfusion with infarct patterns and early neurological outcomes in symptomatic intracranial atherosclerotic stenosis

**DOI:** 10.3389/fneur.2025.1551364

**Published:** 2025-05-29

**Authors:** Xiangming Xu, Linfang Lan, Zhuhao Li, Wenli Zhou, Jing Yang, Xinyi Leng, Yuhua Fan

**Affiliations:** ^1^Department of Neurology, The First Affiliated Hospital of Sun Yat-sen University, Guangzhou, China; ^2^Department of Radiology, The First Affiliated Hospital, Sun Yat-sen University, Guangzhou, China; ^3^Department of Medicine and Therapeutics, Prince of Wales Hospital, The Chinese University of Hong Kong, Hong Kong, Hong Kong SAR, China

**Keywords:** intracranial atherosclerotic stenosis, cerebral perfusion, infarct patterns, neurological outcome, computed tomography perfusion

## Abstract

**Objectives:**

Intracranial atherosclerotic stenosis (ICAS) is a major cause of ischemic stroke, with various infarct patterns. We aimed to investigate the cerebral perfusion features underlying different infarct patterns and the relationship between cerebral perfusion and early neurological outcomes in symptomatic ICAS (sICAS).

**Methods:**

Patients with 50%–99% sICAS in the anterior circulation were enrolled. Cerebral perfusion measures were obtained from computed tomography (CT) perfusion images, including infarct core volumes, penumbra defined with Tmax values > 6 s and > 4 s, and penumbra-core mismatch. Infarct patterns on diffusion-weighted magnetic resonance imaging (MRI) were categorized into four categories: borderzone, perforator, territorial, and mixed patterns. A favorable early neurological outcome was a decrease in the National Institutes of Health Stroke Scale (NIHSS) of ≥1 point at discharge compared with admission.

**Results:**

We recruited 144 patients (median age: 66 years; 61.8% male patients). Significant perfusion compromise was observed in patients with borderzone or territorial infarcts compared to those with perforator infarct patterns. Patients with a favorable early neurological outcome exhibited smaller volumes of penumbra and penumbra-core mismatch at baseline. A multivariate logistic regression analysis revealed that penumbra (defined by Tmax of >4 s)-core mismatch volume of >15 mL was independently associated with a lower chance of achieving a favorable early neurological outcome (adjusted odds ratio, 0.323; 95% confidence interval, 0.121–0.866; *p* = 0.03).

**Conclusion:**

Hemodynamic compromise likely underlies borderzone and territorial cortical/subcortical infarcts in patients with sICAS. The penumbra-infarct core mismatch volume in CT perfusion, with Tmax of >4 s defining the penumbra, was associated with early neurological outcomes of sICAS patients.

## Introduction

Intracranial atherosclerotic stenosis (ICAS) represents a major cause of ischemic stroke and transient ischemic attack, particularly in the Chinese population (1, 2). Perfusion failure and artery-to-artery embolism are standard stroke mechanisms in ICAS (3). It has been reported that hemodynamic compromise and artery-to-artery embolism often interact, particularly in the borderzone regions of middle cerebral artery (MCA) stenosis, where impaired washout of emboli occurs (4, 5). Previous studies have also demonstrated perfusion impairment as an independent predictor for the risk of recurrent stroke in patients with symptomatic ICAS (sICAS). For example, the Vertebrobasilar Flow Evaluation and Risk of Transient Ischaemic Attack and Stroke (VERiTAS) study associated distal blood flow compromise, as measured by quantitative magnetic resonance angiography, with an increased risk of subsequent stroke in patients with sICAS in vertebrobasilar arteries (6).

In patients with extracranial or intracranial arterial occlusive disease, cerebral perfusion has been investigated for a long time using various imaging methods and parameters, such as oxygen extraction fraction in positron emission tomography (7) or cerebral vasoreactivity by transcranial Doppler (8). The above-mentioned earlier methods were unable to quantify the volume of brain tissue with ischemia or at risk of ischemia; however, there have been emerging methods for this purpose in recent years. For instance, the volume of tissue with time to the maximum of residue function (Tmax) of >6 s on computed tomography (CT)/magnetic resonance (MR) perfusion imaging has been used to quantify the penumbra tissue volume in recent studies (9, 10). Moreover, the penumbra-to-infarct core mismatch ratio has been used to gauge the amount of salvageable tissue that may benefit from reperfusion therapy (10). The clinical significance of these quantitative perfusion measures has been more often investigated in studies on hyperacute reperfusion therapy in ischemic stroke. However, there is limited evidence available on ICAS patients. A small-scale study (*n* = 26) found that a mismatch of ≥15 mL between penumbra (defined either by Tmax > 6 s or > 8 s) and infarct core was associated with neurological deterioration at 30 days in patients with anterior-circulation sICAS (11).

In the current study, we aimed to reveal the characteristics of cerebral perfusion in acute ischemic stroke patients with sICAS in the anterior circulation. We used quantitative perfusion measures in CT perfusion (CTP) to investigate their associations with the infarct topology and the early neurological outcomes.

## Methods

### Study design and subjects

This observational study was approved by the First Affiliated Hospital of Sun Yat-sen University Clinical Research Ethics Committee. Written informed consent was waived due to the retrospective study design. Patients with acute ischemic stroke were recruited from January 2017 to May 2022 at the First Affiliated Hospital of Sun Yat-sen University, based on the following inclusion criteria: (1) admission to the stroke unit within 14 days of symptom onset; (2) ischemic stroke attributable to 50–99% atherosclerotic stenosis of the intracranial portion of internal carotid artery (ICA) or M1 segment of MCA, as observed in computed tomography angiography (CTA); and (3) completion of a patient who underwent brain CTP and MRI examinations during hospitalization. Patients were excluded if they met any of the following criteria: (1) concurrent severe stenosis in extracranial arteries; (2) the occurrence of an index ischemic event attributed to non-atherosclerotic intracranial stenosis (e.g., Moyamoya disease, vasculitis, or dissection); (3) evidence of potential cardioembolic stroke (e.g., atrial fibrillation); (4) presence of a known arteriovenous malformation or aneurysm; or (5) unstable vital signs, impaired consciousness, malignant tumors or severe organ dysfunction.

Patient demographics, cardiovascular risk factors, and laboratory test results were collected. The neurological severity of the index stroke was evaluated using the National Institutes of Health Stroke Scale (NIHSS). The location and luminal stenosis of the culprit ICAS were determined based on reconstructed CTA using the warfarin–aspirin symptomatic intracranial disease (WASID) method (12). The severity of stenosis was categorized as moderate (50%–69%) and severe (70%–99%) (13).

### Multimodal CT examination and assessment of cerebral perfusion

All patients underwent a comprehensive CT scan using a 320-detector row, 640-slice cone-beam multidetector CT scanner (Aquilion One, Toshiba Medical Systems, Japan). A whole-brain non-contrast CT scan was performed in a wide-volume mode, with five rotations and a detector width of 4 cm. Following the non-contrast CT scan, CTP was acquired by administering 40 mL of the contrast agent (Ultravist 370, Bayer HealthCare, Berlin, Germany) intravenously at a rate of 5 mL/s, accompanied by 40 mL of saline. The CTP acquisition parameters were as follows: 120 kV, 112 mAs, and a total collimation width of 16 cm. The scan begins at 7 s after the contrast injection, employing a pulsed complete rotation technique with 19 time points acquired over 60 s. The 19 scans were divided into five parts: one scan at 7 s after contrast injection; three, six, and four scans with 2-s cycle time, respectively, starting at 11, 17, and 30 s; and five scans with 5-s cycle time starting at 40 s.

Perfusion parameters, including cerebral blood volume (CBV), cerebral blood flow (CBF), mean transit time (MTT), and Tmax, were automatically measured by the F-STROKE software version 1.0.18 (Neuroblem Ltd., Shanghai, China) in CTP source images. To offset the effects of inter-subject variations in absolute perfusion measures, we used relative perfusion measures in this study. Relative CBF (rCBF) was calculated as the ipsilesional CBF value divided by the CBF value in the contralesional MCA territory. Similarly, we calculated the relative MTT (rMTT) and relative Tmax (rTmax) of the ipsilesional MCA territory. The infarct core was defined as the ipsilesional brain region with CBF of <30%. The penumbra (the tissue at risk of ischemia) was defined with two thresholds in Tmax, that is, the area with Tmax of >6 s and of >4 s in the MCA territory. The penumbra-core mismatch volume was calculated by the penumbra volume minus the infarct core volume. An unfavorable perfusion pattern was defined as a penumbra-core mismatch volume of 15 mL or more.

### Assessment of infarct topology

**Figure 1:**
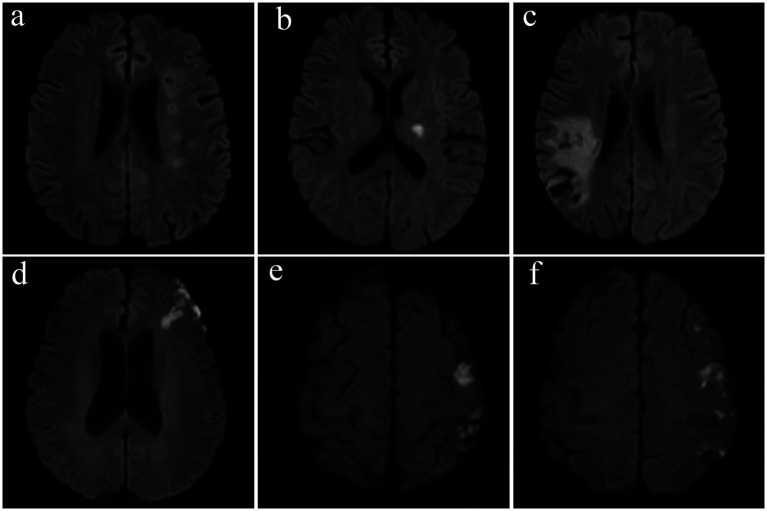
Representative examples of infarct patterns in four patients with symptomatic intracranial atherosclerotic stenosis. **(a)** Borderzone pattern, with chain-like, multiple infarcts in the internal borderzone, **(b)** perforator pattern, **(c)** territorial pattern, with wedge-shaped infarcts involving the cortical and subcortical territory of the inferior branch of the middle cerebral artery, and **(d–f)** a mixed pattern, with external borderzone infarcts shown in subpart **(d)** and multiple cortical infarcts shown in subparts **(e,f)**.

Two investigators (X. Xu and L. Lan) independently assessed and recorded the infarct topology of the index stroke on diffusion-weighted MR imaging, based on the published arterial supply templates (14), categorizing the infarcts into borderzone, perforator, territorial, and mixed patterns (Figure 1). The borderzone pattern referred to infarcts in the internal and external borderzone areas. The perforator pattern was defined with an isolated acute infarct in the penetrating artery territory adjacent to the stenosed intracranial artery. The territorial pattern was described as one or more cortical or subcortical infarct(s) lying entirely in the index diseased intracranial artery, without involvement of the borderzone areas. A mixed pattern refers to the coexistence of two or more of these patterns. In cases of disagreement, the two investigators met and achieved a consensus.

### Outcome measure

The treatment strategy for the index ischemic stroke followed the latest clinical guidelines. A favorable early neurological outcome was defined as a decrease in NIHSS of ≥ 1 point at discharge compared with the score on admission (15), assessed by the neurologist in charge. The independent investigators (X. Xu and J. Yang) assessed the interrater reliability in scoring NIHSS in 20 randomly selected patients. The results showed substantial inter-rater reliabilities with an intraclass correlation coefficient of 0.982 (95% confidence interval [CI]: 0.955–0.993). In consideration of the influence of reperfusion therapies on the perfusion status and patients’ outcomes, we excluded patients who received endovascular or intravenous reperfusion treatment from the analysis of the relationship between cerebral perfusion metrics and the outcome.

### Statistical analyses

Data are presented as medians [interquartile range (IQR) or numbers (percentage)]. For comparison of the variables between groups with different infarct patterns, the Kruskal–Wallis test was used for continuous variables, and Pearson’s chi-squared test or Fisher’s exact method was used for categorical variables. The Benjamini–Krieger–Yekutieli two-stage procedure was used *post-hoc* to control the false discovery rate (FDR), with a *q*-value threshold set to 0.05. A comparison of variables between the groups with or without a favorable early neurological outcome was conducted using the Mann–Whitney U test for continuous variables and Pearson’s chi-squared test or Fisher’s exact method for categorical variables. Binary logistic regression analyses were employed to examine the association between cerebral perfusion measures and the outcome. Odds ratio (OR) and 95% CIs were calculated for all models. In Model 1, adjustments were made for age, NIHSS score at admission, and other variables with a *p*-value of <0.05 in univariate comparisons. Model 2 included the onset to the CTP period based on Model 1. All statistical analyses were performed in SPSS (version 23.0, IBM, Inc., United States). The level of statistical significance was a *p*-value of <0.05 (two-sided).

## Results

### Patients’ characteristics

**Figure 2:**
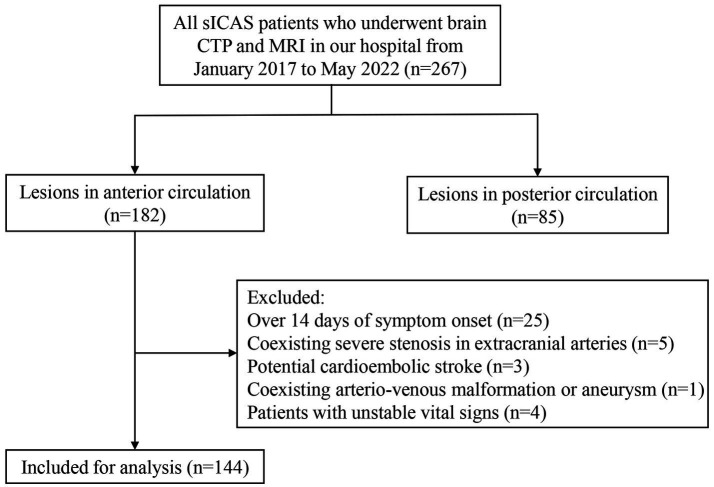
Flowchart of study patient enrollment. sICAS, symptomatic intracranial atherosclerotic stenosis; CTP, computed tomography (CT) perfusion.

A total of 144 acute stroke patients with sICAS were enrolled in the study (Figure 2), with a median age of 66 years and 61.8% being male. The median NIHSS at admission was 4 (IQR: 2–7). The most frequently observed infarct pattern was the mixed pattern, all having mixed borderzone and territorial patterns (*n* = 55; 38.2%), followed by the perforator pattern (*n* = 37; 25.7%). There was a higher percentage of smokers in the territorial and mixed pattern groups. The degree of stenosis in the perforator pattern group was lower than that in other groups. Other baseline characteristics were not significantly different among these groups (Table 1).

**Table 1:** Baseline characteristics of patients with different infarct patterns.

Characteristics	All (*n* = 144)	Borderzone pattern	Perforator pattern	Territorial pattern	Mixed pattern (*n* = 55)	*p-*values
		(*n* = 28)	(*n* = 37)	(*n* = 24)		
Age, years	66 (58–73)	68 (57–75)	66 (57–72)	64 (59–76)	66 (57.5–71.5)	0.944
Male	89 (61.8)	16 (57.1)	17 (45.9)	18 (75)	38 (69.1)	0.066
Vascular risk factors						
Smoking	65 (45.1)	8 (28.6)	13 (35.1)	14 (58.3)	30 (54.5)	0.041
Dyslipidemia	7 (4.9)	0 (0)	2 (5.4)	3 (12.5)	2 (3.6)	0.189
Hypertension	99 (68.8)	22 (78.6)	26 (70.3)	14 (58.3)	37 (67.3)	0.464
Diabetes mellitus	50 (34.7)	11 (39.3)	16 (43.2)	9 (37.5)	14 (25.5)	0.307
History of stroke/TIA	30 (20.8)	10 (35.7)	8 (21.6)	5 (20.8)	7 (12.7)	0.113
History of ischemic heart disease	6 (4.2)	1 (3.6)	2 (5.4)	0 (0)	3 (5.5)	0.837
SBP, mmHg	148 (130–167)	154 (132–175)	150 (134–180)	148 (123–162)	145 (126–163)	0.178
DBP, mmHg	86 (76–96)	85 (78–97)	89 (80–97)	81 (66–93)	85 (74–96)	0.117
Onset to admission, days	2 (1–5)	2 (2–7)	1 (0.5–4)	1.3 (0.38–4)	3 (1–5.5)	0.106
NIHSS at admission	4 (2–7)	4 (2–8)	4 (2–5)	4 (3–8)	4 (3–8)	0.209
NIHSS at admission >4	59 (41)	10 (35.7)	14 (37.8)	10 (41.7)	25 (45.5)	0.818
Laboratory test results						
Fasting Glucose, mmol/L	5.2 (4.6–7.4)	7.2 (4.8–8.2)	6.1 (4.6–7.4)	5.1 (4.6–7.4)	5.1 (4.6–6.4)	0.277
HbA1c, mmol/L	6.1 (5.5–7.7)	6.5 (5.7–8.1)	6.9 (5.5–8)	6.2 (5.5–6.7)	6 (5.4–7.0)	0.454
Triglyceride, mmol/L	1.3 (1–1.9)	1.7 (1.2–2.1)	1.4 (1.1–2.3)	1.1 (0.9–1.6)	1.3 (1–1.8)	0.24
HDL, mmol/L	1 (0.9–1.2)	1 (0.9–1.2)	1 (0.9–1.2)	1 (0.9–1.1)	1 (0.8–1.1)	0.498
LDL, mmol/L	2.8 (2.3–3.5)	3 (2.2–3.5)	2.8 (2.2–3.4)	2.7 (2.3–3.7)	2.7 (2.3–3.5)	0.97
Severity of sICAS						
Degree of stenosis	80 (62–99)	90 (70–99)	70 (50–90)	90 (70–99)	90 (69–99)	0.004
Severe stenosis (70–99%)	108 (75)	23 (82.1)	22 (59.5)	22 (91.7)	41 (74.5)	0.028
Recanalization therapy						
IV rtPA	3 (2.1)	0 (0)	2 (5.4)	0 (0)	1 (1.8)	0.837
EVT	24 (16.7)	7 (25)	4 (10.8)	4 (16.7)	9 (16.4)	0.116
Hospital stays, days	12 (9–14)	12 (8–17)	11 (8–14)	12 (8–15)	12 (9–14)	0.904

Data are median (IQR), or *n* (%). SBP, systolic blood pressure; DBP, diastolic blood pressure; NIHSS, National Institutes of Health Stroke Scale; TIA, transient ischemic attack; HbA1c, hemoglobin A1c; HDL, high-density lipoprotein cholesterol; LDL, low-density lipoprotein cholesterol; sICAS, symptomatic intracranial atherosclerotic stenosis; rtPA, recombinant tissue plasminogen activator; EVT, endovascular treatment.

### Cerebral perfusion features in different infarct patterns

**Figure 3:**
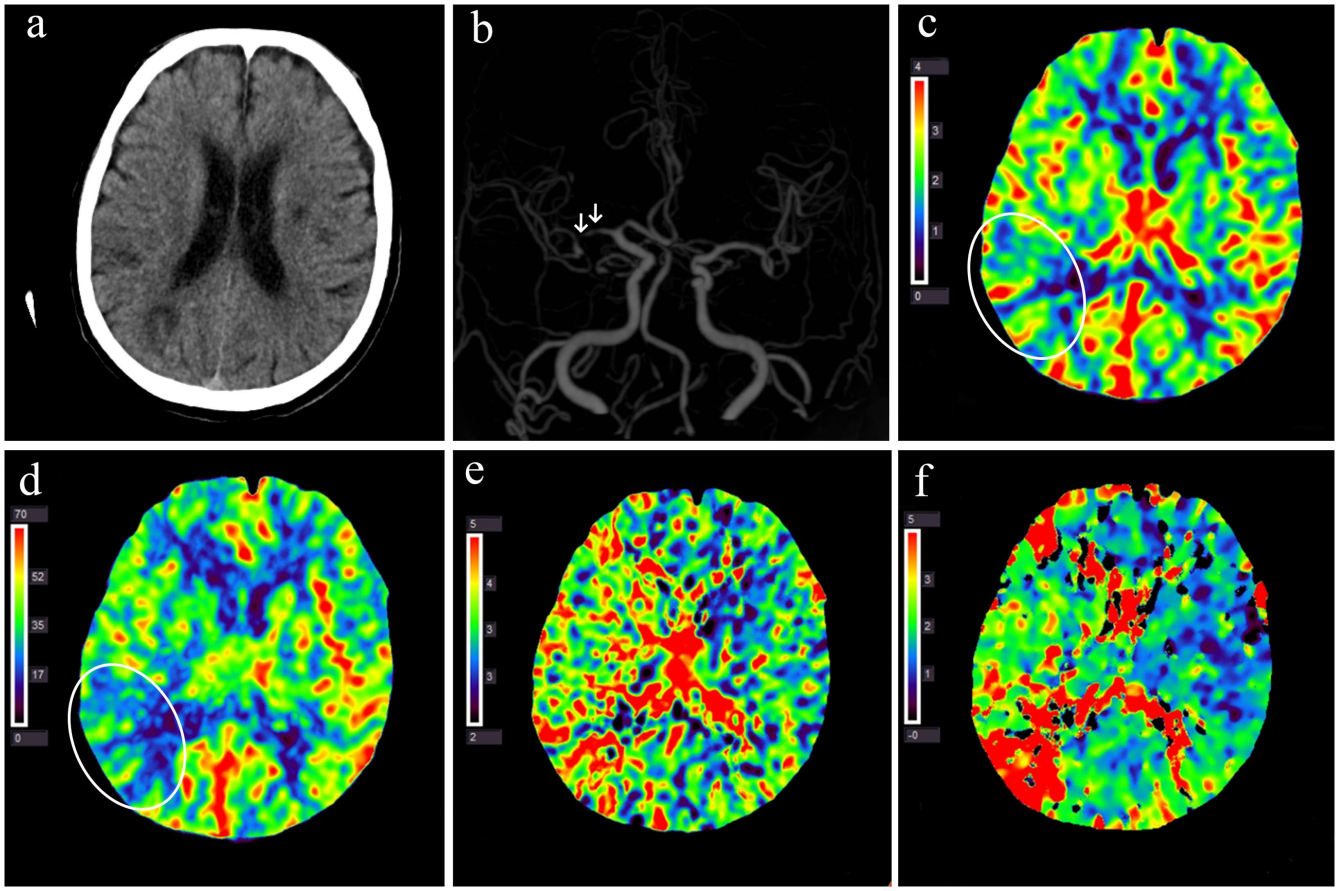
Multimodal CT examination of a patient with acute infarction at the right occipitoparietal junction 1 day after symptom onset. **(a)** Non-contrast CT shows multiple hypodense lesions in the right parieto-occipital lobe and bilateral basal ganglia. **(b)** CT angiography reveals multiple severe stenoses in the M1 segment of the right middle cerebral artery (white arrow). **(c–f)** Perfusion imaging demonstrates decreased CBV **(c)** and CBF **(d)** in the right occipitoparietal junction (white circles) and prolonged MTT **(e)** Tmax **(f)** in the right hemisphere. CBF, cerebral blood flow; CBV, cerebral blood volume; MTT, mean transit time; Tmax, the time to the maximum of residue function.

The median interval between stroke onset and CTP examination was 3 (IQR: 1–7) days. On average, the perfusion time was prolonged (rMTT and rTmax of >1.0), and cerebral blood volume (rCBV > 1.0) was higher in the ipsilesional than the contralateral MCA territory to the sICAS. Figure 3 illustrates the cerebral perfusion characteristics in a patient with a borderzone infarct pattern. Perfusion imaging was able to detect the infarct core in 50 patients, with a median volume of 0 (IQR: 0–0.87) mL. The penumbra-core mismatch volume defined by Tmax of >4 s was 54.3 mL (IQR: 4.5–110.7) and Tmax of >6 s was 1.9 mL (IQR: 0–38.5).

Among patients with different infarct patterns, significant differences were observed in the cerebral perfusion parameters, including rCBV, rMTT, rTmax, infarct core volume, and mismatch volume (Table 2). In pairwise comparisons, the territorial pattern group presented a larger infarct core volume than all other patterns. The penumbra-core mismatch volume, defined by a Tmax delay of >4 s or of >6 s, was significantly smaller in patients with a perforator pattern compared to all other patterns. However, there was no statistically significant difference in penumbra-core mismatch volume between the borderzone and territorial pattern groups.

**Table 2:** Cerebral perfusion in patients with different infarct patterns.

Variable	All (*n* = 144)	Borderzone pattern (*n* = 28)	Perforator pattern (*n* = 37)	Territorial pattern (*n* = 24)	Mixed pattern (*n* = 55)	*p*-values
Onset to CTP, days	3 (1–7)	3 (2–7.8)	2 (0.5–6.5)	2 (0.4–6.8)	4 (1–8)	0.254
rCBF	1 (0.96–1.09)	1.05 (0.96–1.15)	1 (0.97–1.06)	1 (0.94–1.06)	1.02 (0.95–1.09)	0.6
rCBV	1.05 (0.99–1.15)	1.11 (1.01–1.26)	1.01 (0.97–1.09)	1.06 (1.02–1.12)	1.06 (0.99–1.18)	0.027
rMTT	1.05 (1.01–1.10)	1.07 (1.03–1.13)	1.01 (0.97–1.05)	1.08 (1.03–1.12)	1.05 (1.01–1.1)	<0.001
rTmax	1.36 (1.14–1.87)	1.5 (1.27–2.08)	1.2 (1.02–1.35)	1.6 (1.26–1.95)	1.5 (1.16–1.87)	0.001
Infarct core volume, mL	0 (0–0.87)	0 (0–0.25)	0 (0–0)	0.72 (0–7.17)	0 (0–1.04)	<0.001
Penumbra and mismatch defined with Tmax > 4 s						
Penumbra volume, mL	55.9 (4.6–112.2)	93.9 (41.1–142.8)	2.5 (0.5–19.4)	81.8 (50–111.1)	68.7 (12.8–124.2)	<0.001
Mismatch volume, mL	54.3 (4.5–110.7)	93.8 (40.5–142.2)	2.5 (0.5–19.4)	69.9 (44.4–107)	62.4 (12.8–124.2)	<0.001
Unfavorable perfusion pattern	94 (65.3%)	23 (82.1%)	9 (24.3%)	22 (91.7%)	40 (72.7%)	<0.001
Penumbra and mismatch defined with Tmax of >6 s						
Penumbra volume, mL	2.2 (0–45.2)	9.6 (0.2–63.3)	0 (0–0.05)	30.6 (1.3–57.9)	5.6 (0.1–54.8)	<0.001
Mismatch volume, mL	1.9 (0–38.5)	9.6 (0.2–63.3)	0 (0–0.05)	19.7 (1.2–44.4)	5.6 (0–54.5)	<0.001
Unfavorable perfusion pattern	53 (36.8%)	13 (46.4%)	3 (8.1%)	14 (58.3%)	23 (41.8%)	<0.001

Data are median (IQR), or *n* (%). CTP, CT perfusion; rCBF, relative cerebral blood flow; rCBV, relative cerebral blood volume; rMTT, relative mean transit time; rTmax, relative time to the maximum of residue function.

### Cerebral perfusion and early neurological outcomes

As mentioned above, the 27 patients who received endovascular or intravenous reperfusion treatment were excluded from the analysis due to the relationship between cerebral perfusion metrics and the outcome, resulting in a total of 117 patients in such analyses. The median interval between the two assessments of the NIHSS (at admission and discharge) was 11 (IQR: 9–14) days. Among 117 patients, 71 (60.7%) demonstrated a favorable early neurological outcome. The baseline data and cerebral perfusion parameters were compared between those with the outcome and those without the outcome (Table 3). Patients’ characteristics at baseline were not significantly different between the two groups. Notably, patients with a favorable early neurological outcome had less severe luminal stenosis in the sICAS lesion (medians 80% vs. 88%; *p* = 0.038). Regarding the cerebral perfusion parameters, no significant difference was observed in rCBV, rCBF, rMTT, rTmax, or infarct core volume between the two groups. However, the penumbra volume and penumbra-core mismatch volume were smaller, with fewer patients having an unfavorable perfusion pattern, defined with Tmax of >4 s or of 6 s, in patients with a favorable early neurological outcome than in those without.

**Table 3:** Characteristics of patients with and without a favorable early neurological outcome.

Characteristics	Favorable early neurological outcome	Otherwise (*n* = 46)	*p-*values
	(*n* = 71)		
Age, years	65 (58–72)	67 (59–76)	0.401
Male sex	43 (60.6)	30 (65.2)	0.612
Vascular risk factors			
Smoking	30 (42.3)	24 (52.2)	0.293
Dyslipidemia	1 (1.4)	4 (8.7)	0.077
Hypertension	45 (63.4)	35 (76.1)	0.149
Diabetes mellitus	29 (40.8)	14 (30.4)	0.254
History of stroke/TIA	13 (18.3)	11 (23.9)	0.463
History of ischemic heart disease	1 (1.4)	3 (6.5)	0.298
SBP, mm Hg	150 (134–171)	151 (132–167)	0.953
DBP, mm Hg	88 (77–97)	88 (78–97)	0.643
Onset to admission, days	2 (1–5)	2 (1–6)	0.658
NIHSS at admission	4 (3–7)	3 (2–6)	0.105
Laboratory test			
Fasting glucose, mmol/L	5.3 (4.6–7.4)	5.2 (4.7–7.8)	0.526
HbA1c, mmol/L	6.2 (5.5–7.7)	6.1 (5.4–7)	0.559
Triglyceride, mmol/L	1.3 (1.0–2.1)	1.3 (1.1–1.8)	0.689
HDL, mmol/L	1 (0.9–1.2)	1 (0.8–1.1)	0.273
LDL, mmol/L	2.9 (2.3–3.7)	2.7 (2.2–3.5)	0.335
Severity of the sICAS			
Degree of stenosis	80 (58–90)	88 (68–99)	0.038
Severe stenosis (70–99%)	48 (67.6)	36 (78.3)	0.211
Hospital stays, days	11 (9–14)	11 (8–14)	0.754
Onset to CTP, days	3 (1–6)	5 (2–8.5)	0.084
rCBF	1.02 (0.97–1.09)	0.99 (0.96–1.07)	0.269
rCBV	1.05 (0.99–1.15)	1.04 (0.97–1.11)	0.584
rMTT	1.05 (1.00–1.08)	1.03 (1.00–1.10)	0.951
rTmax	1.2 (1.06–1.69)	1.4 (1.2–1.76)	0.142
Infarct core volume, mL	0 (0–0.27)	0 (0–0.57)	0.347
Penumbra and mismatch defined with Tmax of >4 s			
Penumbra volume, mL	23.2 (1.4–103)	76.5 (25.8–109.6)	0.041
Mismatch volume, mL	23.2 (1.4–100.5)	66.1 (25.8–100.2)	0.037
Unfavorable perfusion pattern	37 (52.1)	35 (76.1)	0.009
Penumbra and mismatch defined with Tmax > 6 s			
Penumbra volume, mL	0.13 (0–25.9)	5.8 (0.2–46.7)	0.012
Mismatch volume, mL	0.04 (0–18.8)	5.8 (0.01–39.1)	0.015
Unfavorable perfusion pattern	18 (25.4)	20 (43.5)	0.041

Data are median (IQR), or *n* (%). SBP, systolic blood pressure; DBP, diastolic blood pressure; NIHSS, National Institutes of Health Stroke Scale; CTP, CT perfusion; TIA, transient ischemic attack; HbA1c, hemoglobin A1c; HDL, high-density lipoprotein cholesterol; LDL, low-density lipoprotein cholesterol; rCBF, relative cerebral blood flow; rCBV, relative cerebral blood volume; rMTT, relative mean transit time; rTmax: relative time to the maximum of residue function.

A multivariate logistic regression analysis revealed that an unfavorable perfusion pattern defined with Tmax of >4 s was independently associated with lower odds of favorable early neurological outcomes, after adjusting for age, baseline NIHSS, stenosis degree of the sICAS lesion, and the onset to CTP time period (adjusted OR: 0.323; 95% CI: 0.121–0.866; *p* = 0.03). The unfavorable perfusion pattern defined with Tmax of >6 s was not significantly associated with the outcome (Table 4).

**Table 4:** Results of multivariate logistic regression analysis on cerebral perfusion parameters in predicting a favorable early neurological outcome.

Variables	Model 1^*^		Model 2^†^	
	Adjusted OR (95% CI)	*p*	Adjusted OR (95% CI)	*p*
Mismatch volume defined by Tmax of >4 s, mL	0.993 (0.985–1.002)	0.13	0.993 (0.984–1.002)	0.12
Unfavorable perfusion pattern defined with Tmax of >4 s	0.354 (0.136–0.923)	0.03	0.323 (0.121–0.866)	0.03
Mismatch volume defined by Tmax of >6 s, mL	0.997 (0.983–1.011)	0.68	0.997 (0.983–1.012)	0.71
Unfavorable perfusion pattern defined with Tmax of >6 s	0.505 (0.203–1.258)	0.14	0.493 (0.195–1.248)	0.14

OR, odds ratio; CI, confidence interval. ^*^Model 1: Multivariable binary logistic regression model after adjusting for age, NIHSS score at admission, and stenosis degree of the sICAS lesion. ^†^Model 2: Model 1 after adjusting for the onset of the CTP time period.

## Discussion

This study revealed various cerebral perfusion characteristics among those with different infarct patterns in patients with acute ischemic stroke due to ICAS. Perfusion compromise was more likely to be observed in those with borderzone infarcts or territorial cortical/subcortical infarcts than in those with a single subcortical infarct in penetrating artery territory. Smaller volumes of penumbra (defined by Tmax > 4 s) and penumbra-infarct core mismatch at baseline were associated with a higher probability of achieving a favorable early neurological outcome upon discharge. This study reinforced cerebral perfusion compromise as an essential pathophysiological mechanism of stroke in ICAS, which may contribute to both borderzone and territorial infarcts and affect early outcomes of the patients.

The relationship between impaired perfusion detected by non-invasive imaging and infarct patterns in patients with large artery occlusive disease has been investigated in previous studies (16–18). However, these earlier studies primarily focused on a specific infarct pattern, while data regarding the perfusion measures in those with different infarct patterns among patients with sICAS are scarce. This study found that the penumbra-core mismatch volume, with the penumbra defined by the brain tissue with Tmax of >4 s or a Tmax delay of >6 s, was the smallest in the perforator infarct pattern group. This finding agrees with those of previous studies suggesting that a single subcortical infarct in the penetrating artery territory, in the presence of ICAS, represents a focal disease associated with the occlusion of a branch by the ICAS lesion, without global perfusion impairment (19, 20). In contrast, patients with borderzone infarct pattern displayed the most significant hemodynamic compromise in CTP in this study, aligning with previous studies that have strongly associated internal borderzone infarctions to a hemodynamic mechanism (16). Another interesting finding of this study was the comparable penumbra and penumbra-core mismatch volumes in patients with territorial and borderzone infarct patterns. Artery-to-artery embolism due to a vulnerable atherosclerotic plaque’s rupture has been generally considered an underlying mechanism of territorial cortical/subcortical infarcts in sICAS (21). This study provides additional evidence supporting the theory that hypoperfusion impairs the ability to clear small emboli stranded in distal territories (22). Hence, the affected patients may be more likely to have territorial infarcts (23).

Few studies investigated the relationships between cerebral perfusion metrics and early neurological outcomes in sICAS patients. In this study, univariate analyses demonstrated that patients with a smaller penumbra and a smaller penumbra-core mismatch volume, defined by Tmax of >4 s and of >6 s, were more likely to have a favorable early neurological outcome. Further multivariate logistic regression analysis showed that an unfavorable perfusion pattern defined with Tmax of >4 s, but not of >6 s, was independently associated with a favorable early neurological outcome after adjusting for age, baseline NIHSS, stenosis degree of the sICAS lesion, and the onset to CTP period. The finding differs from that of a previous study (*n* = 26), as mentioned above, which reported that a penumbra (defined using Tmax of >6 s or of >8 s)-core mismatch volume of >15 mL was associated with neurological deterioration at 30 days, but not when Tmax of >4 s was used in defining the penumbra (11). The inconsistency of the study findings could be attributed to various study populations (Chinese vs. mostly Caucasians), different sample sizes, or the more significant perfusion impairment in patients involved in the previous study (e.g., the median mismatch volumes defined by Tmax of >4 s in the two groups in this study were 23.2 and 66.1 mL; however, the median in the two groups in the previous study were 16.6 and 120 mL). In another recent study of sICAS patients (*n* = 107), the volume of the median Tmax of >6 s was 0 mL (IQR: 0–15) before angioplasty ± stenting therapy (comparable with the current study with a median of 2.2 mL), which was 0 (IQR: 0–0) after the endovascular treatment. However, the volume of the median Tmax of >4 s changed from 74 (IQR: 28–178) to 22 mL (IQR: 0–101) with angioplasty ± stenting therapy. Therefore, the study concluded that the volume of Tmax of >4 s may be more suitable than Tmax of >6 s to quantify perfusion compromise in sICAS patients (24). As these perfusion metrics were derived from hyperacute reperfusion therapy studies, further studies are needed to explore the optimal metrics and thresholds in assessing the perfusion status and predicting the outcomes in sICAS patients.

Our study provides valuable insights into the significance of penumbra-core mismatch on CT perfusion among patients with sICAS. We found that perfusion mismatch was associated not only with the borderzone infarct pattern but also with the territorial infarct pattern. Furthermore, a larger mismatch volume (defined by Tmax > 4 s) was less likely to be associated with a favorable early neurological outcome in sICAS patients. These findings underscore the significance of cerebral perfusion compromise in the risk stratification of sICAS patients and may further inform individualized treatment and secondary prevention. For instance, treatment strategies to restore cerebral perfusion, such as angioplasty ± stenting, may benefit patients with sICAS and hemodynamic compromise, which warrants further studies.

There were some limitations to this study. First, only patients with anterior-circulation stroke were included, as the infarct patterns in the posterior circulation differed and thus were challenging to group alongside anterior-circulation infarcts. Moreover, as mentioned above, the perfusion metrics measured in this study were mostly adopted from previous studies on hyperacute reperfusion therapy in ischemic stroke; it is unclear whether they are applicable or reliable in assessing the perfusion status in sICAS. The small infarct core and Tmax of >6 s volumes, as quantified in this and previous studies (11), may imply underestimation of the perfusion impairment based on these metrics in sICAS patients, which warrants validation with other imaging methods. Another limitation was that we used NIHSS reduction of ≥1 in defining the favorable early neurological outcome, with a cohort of patients with relatively mild neurological deficits (median admission NIHSS = 4; IQR: 2–7), but we demonstrated high inter-rater reliability in assessing the NIHSS score in this cohort. Furthermore, data were unavailable for the relationship between the cerebral perfusion metrics and long-term outcomes of the sICAS patients in this study, which warrants further investigation.

In conclusion, this study reinforced cerebral perfusion impairment as an important pathophysiological mechanism of stroke that was associated with borderzone and territorial cortical/subcortical infarcts in patients with sICAS. The penumbra-infarct core mismatch region in CTP, using Tmax of >4 s to define the penumbra, may represent the brain tissue at risk of ischemia in sICAS patients. This penumbra-core mismatch was associated with early neurological outcomes of sICAS patients.

## Data Availability

The raw data supporting the conclusions of this article will be made available by the authors upon reasonable request.
